# Examining the incentive effects of athlete compensation: a combined analysis using SEM and fsQCA

**DOI:** 10.3389/fpsyg.2024.1375088

**Published:** 2024-10-21

**Authors:** Huan Zhao, Hebin Chong, Feiyan Xiao, Li Tan, Zhaoxia Liu

**Affiliations:** ^1^School of Physical Education, Shandong Youth University of Political Science, Jinan, China; ^2^School of Sports Science, Honam University, Gwangju Metropolitan City, Republic of Korea; ^3^Faculty of Physical Education, Tomsk State University, Tomsk, Russia; ^4^School of Physical Education, Hunan Agricultural University, Changsha, China

**Keywords:** athlete, compensation incentive effect, compensation structures, structural equation model, fsQCA

## Abstract

In the context of competitive sports, the evaluation of compensation incentive effectiveness is key to the optimization of compensation systems for athletes. This study creates a model of the athlete compensation incentive effect from single and multinomial grouping perspectives, combining empirical research (SEM) with fuzzy-set qualitative comparative analysis (fsQCA) based on a sample of 352 validated data. The results lead to two findings. First, athletes’ direct economic compensation, direct non-economic compensation, and indirect non-economic compensation have a significant positive effect on the incentive effect of compensation. Second, that the incentive effect of high compensation has two configurations, namely “economic value” and “economic environmental value.” It seems that the effective combination of compensation factors can enhance the motivation effect in a “different way.”

## 1 Introduction

Athlete motivation is one of the most important factors influencing the quality development of competitive sports. Motivation is dependent upon an individual’s expectations about their ability to perform tasks and obtain the desired rewards (Yavuz, 2004). To motivate athletes into training, the state has adopted a series of measures, such as the formation of a remuneration structure of “basic salary & training allowance & bonus and subsidy” in terms of financial compensation. This can be a fair reward for athletes’ hard work and good performance, but also an important way of motivating more people to actively participate in sports. In addition to financial compensation, the government also provides career development opportunities and educational support for outstanding athletes who can participate in high-level training and have access to higher education. Economic and non-economic compensation together constitute a perfect system of compensation system for athletes. Through this approach, the system can implement a comprehensive set of incentives to achieve more effective motivation for athletes. However, we will need to think if based on the development of athletic needs, the existing compensation incentives are effective. We would also need to consider how to optimize the incentives to achieve the optimal effect under the condition of limited resources that deserve attention and further research.

The incentive effect is a complex dynamic system, and the interaction iteration of different compensation elements will produce different incentive effects. Currently, scholars have researched the impact of various elements of compensation on the motivational effects of athletes. Chan et al. (2009) confirm that prize money has been significantly used to motivate athletes to win.

Their study concluded that non-economic compensation, by providing athletes with tangible symbols of achievement (Yavuz, 2004) serves as an effective complement to monetary incentives, motivating athletes to aim for medal-winning performances (Tshube et al., 2012). Zhao et al. (2022) found that direct economic compensation, direct non-economic compensation, and indirect non-economic compensation have a significant positive effect on athletes’ compensation incentive effect. Scholars have proposed different compensation incentives and also analyzed the effects of individual factors, but few studies have examined the multiple interactions of incentive factors on the effectiveness of athletes’ incentives from a general perspective.

This paper adopts a research method that combines structural equation modeling (SEM) and fuzzy-set qualitative comparative analysis (fsQCA), integrating multiple factors of compensation, and exploring their influence on athletes’ compensation incentives and their factor combination mechanisms. In the first stage, SEM structural equation modeling is used to analyze the specific influence paths and path coefficients of each compensation factor on athletes’ incentive effects, trying to reveal the “net effect” of each compensation factor on incentive effects. In the second stage, the fsQCA method is used to further study the “combination effect” of factors with influence on athletes’ motivation effect by applying configuration thinking. We aim to answer the question, “What factors influence the incentive effect of athletes’ compensation?” and “How does the combination of these factors affect the effectiveness of athletes’ compensation incentives?” to provide theoretical guidance and management insights for optimizing athletes’ compensation structure.

## 2 Literature review and hypotheses development

Through the research and discussion of local and international scholars on compensation, it is found that the vocabulary used by academics has roughly gone through the process from “Wage to Salary,” and then to the Compensation (Tan et al., 2019). It has also been observed that it has evolved from a single monetary, or in-kind compensation (Peng, 2004), to the rewards in the monetary or non-monetary form provided to employees by companies in exchange for their time, skills, efforts, and achievements (WorldatWork, 2021). Therefore, this study further clarifies that the form of compensation defined in the evaluation of compensation incentive effect for professional athletes is comprehensive compensation, which includes four forms direct economic compensation, indirect economic compensation, direct non-economic compensation, and indirect non-economic compensation (Zhao et al., 2022). The “compensation incentive effect” reflects the employee’s efforts on the various types of compensation, including two kinds of compensation incentives: feelings (job satisfaction) and willingness to work more (Jiang and Du, 2014).

Social exchange theory asserts that all human behavior is governed by some kind of exchange activity that brings rewards and incentives (Blau, 1964). Further research on this theory has demonstrated the important role of total compensation in influencing employees’ attitudes and behaviors (Alhmoud and Rjoub, 2019). It is argued that employees’ attitudes and positive behaviors are always guided by the resources available to them at work (Ji and Cui, 2021) and that when individuals receive resources from the organization, they develop positive feelings about the organization and its values, and thus, are more willing to work hard to achieve organizational goals (He et al., 2014). The overall reward system, as an effective work resource program, directly affects employee engagement and satisfaction (Bakker et al., 2014; Narang and Sharma, 2018). Based on the above theoretical and empirical studies, the following theoretical hypotheses are thus proposed:

H1: Athletes’ direct economic compensation positively influences compensation incentive effects.

H2: Athletes’ indirect economic compensation positively influences compensation incentive effects.

H3: Athletes’ direct non-economic compensation positively influences compensation incentive effects.

H4: Athletes’ indirect non-economic compensation positively influences compensation incentive effects.

Empirical research on athletes, has identified key personality traits, including patriotism, development orientation, hedonism, aggressiveness, and cooperation. These traits play a significant role in shaping athletes’ aspirations for competitive remuneration and benefits. Athletes are motivated by their professional commitment to contribute to a national pursuit of glory in sporting events. Furthermore, they actively seek opportunities for personal development and place importance on fostering harmonious interpersonal communication. This insight emerges from an academic examination of the subject. The diversity of demand characteristics determines the comprehensiveness of athletes’ compensation incentive structure. In the daily regular management practice of athletes, economic and non-economic compensation generally exist at the same time, and the most ideal scenario indicates that the two interact synergistically to enhance the effectiveness of compensation incentives. Based on this, the following research hypotheses are proposed:

H5: The incentive effect of athlete compensation is the result of the interaction and combination of multiple incentives, and many different combinations of paths lead to this result.

**Figure 1:**
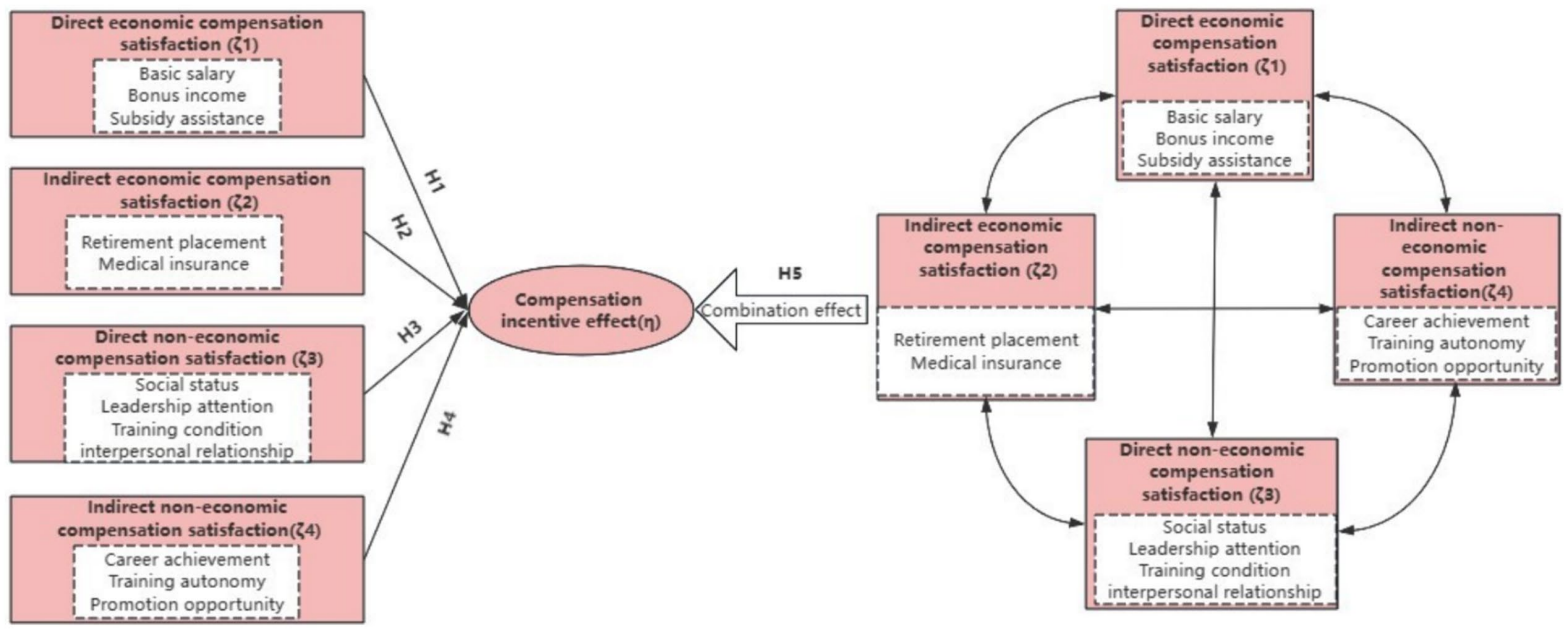
Model of the research hypothesis.

Based on the research hypotheses proposed in this study, the elemental structure of comprehensive remuneration will be integrated, and the research method combining structural equation modeling (SEM) and fuzzy set qualitative comparative analysis (fsQCA) will be used to explore in-depth the relationship between the 12 antecedent conditions of the four dimensions, as shown in Figure 1.

## 3 Research process and methods

### 3.1 Questionnaire design

The questionnaire was designed based on scientific, systematic, easily accessible, and non-oriented principles and was combined with changes in the salary structure of athletes in China. The questionnaire consisted of two parts: (a) basic personal information and (b) athletes’ perceptions and satisfaction with their salaries. In the first part, basic personal information includes name, gender, age, training years, level, program, monthly income, and region. According to relevant sources (Chen, 2010; Lazear, 2018; Wen, 2006), the second part contains 14 items of satisfaction measurement. A five-point Likert scale was used to measure athletes’ salary satisfaction, according to which “1” equals “strongly dissatisfied,” “2” equals “somewhat dissatisfied,” “3” equals “neither satisfied nor dissatisfied,” “4” equals “somewhat satisfied,” and “5” equals “strongly satisfied.”

### 3.2 Data collection and processing

A self-report questionnaire was used to collect data for this study. According to the Declaration of Helsinki, all subjects gave written informed consent. Survey subjects were assured confidentiality and anonymity. All participation was voluntary. Ethical approval was provided by the Beijing Sport University Ethics Committee of Sport (Project number: 2021124H). This study was conducted in accordance with relevant guidelines and regulations.

To ensure the recovery rate and efficiency of questionnaires, they were distributed and collected at the Chenggong Training Base and Ridge Training Base in Kunming, Yunnan, China, Ersha Sports Training Center, and CBA Training Venue in Guangzhou, Guangdong, China. A total of 400 questionnaires were distributed, out of which 390 were collected. Among them, 38 invalid questionnaires were excluded, and 352 valid questionnaires were kept, with a recovery rate of over 90%. The sample size was 5–10 times the number of items, which should be about 130, since there were 13 items in this study. G*power calculations resulted in a sample size of 134 which was enough for this study. Due to the regional differences in China’s economic development level, athletes from Guangdong, Liaoning, Hunan, Jilin, Gansu, and Yunnan provinces, representing the eastern, central, and western regions, were selected as the subjects of the survey. The information distribution of the samples is shown in Table 1. The distribution of sample objects seems reasonable.

**Table 1:** Characteristics of the sample.

Basic information	Category	Frequency	%
Gender	Male	213	60.5
	Female	139	39.5
Age	≤18	53	15.1
	19–24	235	66.7
	≥25	64	18.2
Training years	≤2	93	26.4
	2–4	120	34.1
	4–6	81	23
	≥6	58	16.5
Level	Master sportsman	158	44.9
	National-level athletes	161	45.7
	Second-level athletes	33	9.4
Monthly income	≤2,000	59	16.8
	2,001–4,000	235	66.7
	4,001–6,000	12	3.4
	>6,000	46	13.1
Region	Eastern region	255	72.4
	Central region	38	10.8
	Western region	59	16.8

### 3.3 Research methods

#### 3.3.1 Structural equation modeling

Structural equation modeling (SEM) is a more flexible approach than linear modeling since with this method we can address causal relationships that are not available in correlation analysis, as well as make a distinction between direct and indirect effects. Therefore, this approach has been successfully applied to human resource management (Coudounaris et al., 2020; Shah et al., 2017), to study job satisfaction (Lu et al., 2017), intention to remain (Shi et al., 2022), compensation satisfaction (Jawahar and Stone, 2011), and compensation incentive effects (Zhao et al., 2022) with better results. Therefore, using AMOS 24.0 software to construct structural equation models helps to explore the relationship between total compensation and incentive effects. Therefore, the following explanatory model was created, by using athletes’ total compensation as an explanatory variable for the incentive effect.

#### 3.3.2 Fuzzy set qualitative comparative analysis

The fuzzy set qualitative comparative analysis (fsQCA) method is based on set theory that considers the research object as a grouping of multiple antecedent variables in different combinations and reveals the complex causal relationships among multiple dependent variables by analyzing the interdependence, group equivalence, and causal asymmetry among the condition variables. The athlete compensation incentive effect as a complex phenomenon may have multiple concurrent causality problems with the interdependence of multiple antecedent variables. Therefore, QCA 3.0 software was used to further explore and analyze the aforementioned antecedent variables using the fsQCA method.

## 4 Analyses and results

### 4.1 Structural model results

According to the first step of the research process, SPSS22.0 software was used to conduct the Kaiser–Meyer–Olkin (KMO) and Bartlett’s sphericity test on the data of each variable of athletes’ compensation incentive effect, where KMO = 0.786 and *p* < 0.001. The results indicate that there is a significant correlation between the evaluation indicators of athletes’ compensation incentive effect evaluation, which is suitable for factor analysis. Among them, it was found that the common degree of the interpersonal relationship (X9) was 0.344 < 0.7 when extracting the common factor for the measurement indicators of athletes’ compensation incentive effect. Thus, the variable was deleted. Finally, an evaluation index system of athletes’ compensation incentive effect with five latent variables and 13 measurement indicators was formed. Furthermore, the questionnaire was tested for reliability, i.e., Cronbach’s Alpha coefficient, and the composite reliability (CR) should be analyzed where these values should be greater than 0.7 and 0.6, respectively. After validation, it was found that both Cronbach’s Alpha coefficient and CR value were greater than 0.7. This indicates that the scale designed in this paper has good reliability. Then, factor loadings (FL) and average variance extracted (AVE) were used to determine the structural validity of the questionnaire, and these values should be higher than 0.6 and 0.5, respectively. The validation data showed that the factor loadings of the variables in the article as well as AVE were higher than the standard values, indicating good convergent validity of the questionnaire. The specific data are shown in Table 2.

**Table 2:** Results of confirmatory factor analysis.

Variable	FL
ζ_1_ (CA = 0.768; CR = 0.795; AVE = 0.568)	
Basic salary (X_1_): Are you satisfied with your current basic salary?	0.600
Bonus income (X_2_): Are you satisfied with the current bonus income?	0.825
Subsidy assistance (X_3_): Are you satisfied with the current subsidy assistance?	0.814
ζ_2_ (CA = 0.735;CR = 0.767;AVE = 0.631)	
Retirement placement (X_4_): Are you satisfied with the current retirement placement policy?	0.937
Medical insurance (X_5_): Are you satisfied with the current medical insurance?	0.620
ζ_3_ (CA = 0.836;CR = 0.837;AVE = 0.631)	
Social status (X_6_): Are you satisfied with the current recognition of athletes’ social status?	0.828
Leadership attention (X_7_): Are you satisfied with the importance your leaders place on you or the team?	0.788
Training condition (X_8_): Are you satisfied with the training environment and conditions?	0.765
ζ_4_(CA = 0.880;CR = 0.884;AVE = 0.717)	
Career achievement (X_10_): How fulfilling is the project you are currently working on?	0.825
Training autonomy (X_11_): Do leaders or coaches value your training ideas?	0.836
Promotion opportunity (X_12_): Do you see a future for your sports team?	0.879
η (CA = 0.834; CR = 0.837; AVE = 0.720)	
Salary incentive feeling (Y_1_): Do you think the current income level motivates athletes?	0.797
Effort will (Y_2_): Are you willing to train hard to achieve results for yourself and your team?	0.897

CA, Cronbach’s alpha; CR, Composite reliability; AVE, Average variance extracted; FL, Factor loading.

**Figure 2:**
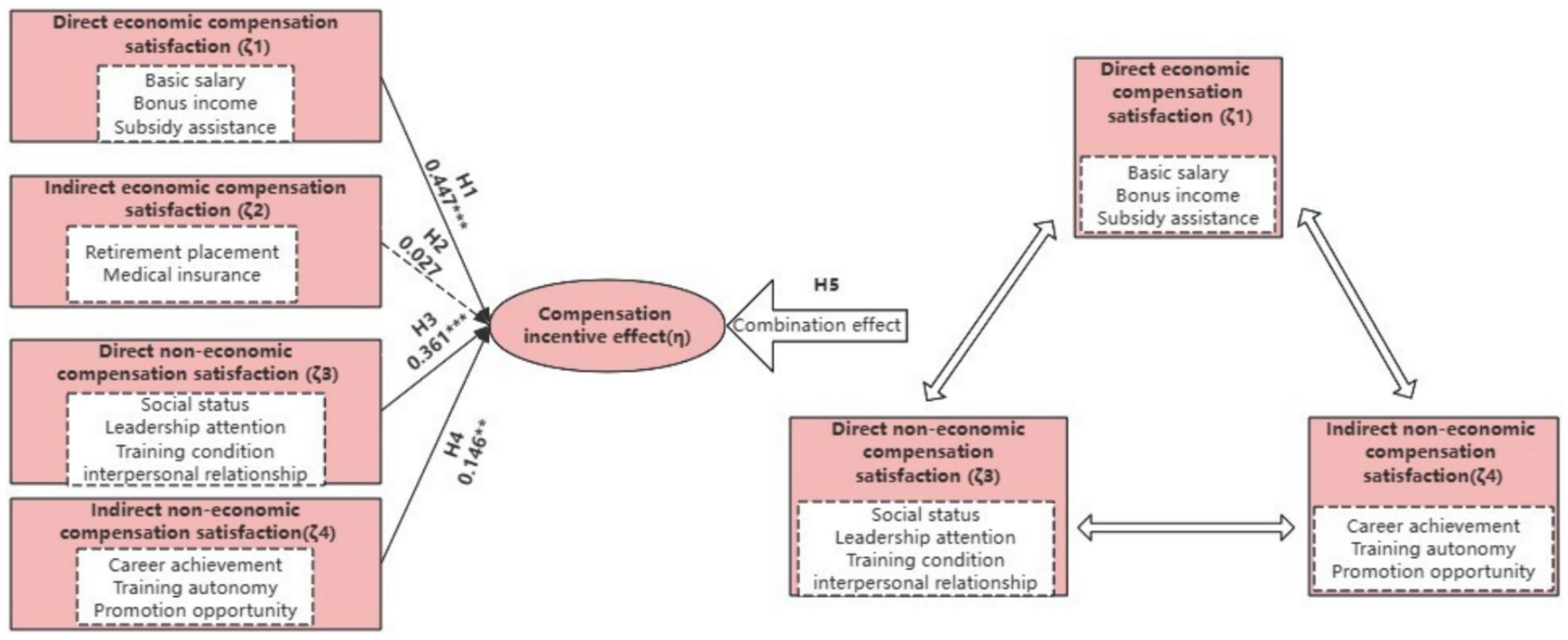
FsQCA analysis model.

Finally, the SEM method was used to provide an estimate of the model shown in Figure 2. From the model fit, the ratio of the chi-square to the degrees of freedom (χ^2^/df) is 1.875 < 3, the normed fit index (NFI) is 0.945 > 0.8, the comparative fit index (CFI) value is 0.974 > 0.9, the goodness of fit index (GFI) is 0.953 > 0.9, and the root mean squared error of approximation (RMSEA) is 0.05 < 0.08. This indicates that the overall fit of the model is good, therefore the theoretical model and the data obtained in this study can be fit. From the path coefficients, all the paths seem to be significant, except indirect economic compensation satisfaction. Therefore, only hypothesis H2 is excluded, and the rest of the hypotheses are valid.

### 4.2 FsQCA results

The results of the SEM described above show that direct economic compensation (ζ1), direct non-economic compensation (ζ3), and indirect non-economic compensation (ζ4) have a significant impact on athlete incentive effects, with direct economic compensation having the greatest impact. However, SEM can only explain the net effect of these three factors on the compensation incentive effect, but not the complex causal relationship between these three factors that are interdependent and work together (combination effect analysis). Therefore, in this study, fsQCA analysis selects three latent variables (ζ1, ζ3, and ζ4) and nine observed variables (X1, X2, X3, X6, X7, X8, X10, X11, and X12) as antecedent variables, and compensation incentive effect as the outcome variable, to analyze the combined effect of each factor on pay incentive effect (see Figure 2).

#### 4.2.1 Data calibration

Calibration of the data for the variables involved in the study is required when applying the fuzzy-set qualitative comparative analysis method, which helps to improve the interpretability of the results. In this study by employing fsQCA 3.0 software, we calibrated the data of each variable according to the criteria proposed by Ragin (2009), 5, 50, and 95%. Nevertheless, cases with an affiliation of 0.5 are removed in the fsQCA analysis (Ragin, 2008). To overcome this problem, this study only modified the value of affiliation from 0.5 to 0.501 (Campbell et al., 2016).

#### 4.2.2 Necessity analysis

It is required to test the necessity of individual antecedent variables in the fsQCA analysis, using coverage and consistency to estimate whether the antecedent variables constitute sufficient and necessary conditions for the outcome variable. When the coverage is greater than 0.8, then the corresponding antecedent variable is an adequate condition leading to the outcome. With consistency greater than 0.9, it is indicated that the antecedent variable is a necessary condition for the outcome variable (Ragin, 2009). As shown in Table 3, the consistency and coverage of the antecedent variables affecting the incentive effect of athletes’ compensation are both less than 0.8, which indicates that for this study none of the antecedent variables can affect and, thus determine, the outcome variable alone but need to be combined to influence the occurrence of the outcome, and therefore a conditional combination analysis of the constructs among the antecedents is required (Zhang and Liu, 2023).

**Table 3:** Results of necessity test.

Conditions tested	Consistency	Coverage	Conditions tested	Consistency	Coverage
X1	0.713	0.759	~X1	0.551	0.559
X2	0.688	0.739	~X2	0.638	0.642
X3	0.775	0.717	~X3	0.539	0.637
X6	0.707	0.745	~X6	0.592	0.606
X7	0.609	0.769	~X7	0.693	0.611
X8	0.613	0.768	~X8	0.684	0.607
X10	0.609	0.762	~X10	0.678	0.602
X11	0. 658	0.727	~X11	0.618	0.606
X12	0.704	0.741	~X12	0.622	0.637

∼, absence of condition.

#### 4.2.3 Configuration analysis

After calibration of all causal conditions and the outcome of interest, this study constructs the truth table. We followed the recommendation that the frequency threshold should be set to cover at least 80% of the study sample (Fiss, 2011; Ragin, 2006). Therefore, based on the number of cases in this study, the frequency threshold was set to 3, the consistency threshold was set to 0.8, and the threshold for the proportional reduction of inconsistency (PRI) was set to 0.75 (Misangyi and Acharya, 2014). Following the construction of the truth table for the standard analysis, the software generates three solutions depending on how the “logical residuals” in the truth table are included in the analysis of the results: complex, parsimonious, and intermediate solutions (Eng and Woodside, 2012; Fiss, 2011). A common way to interpret the results of the analysis is to use the intermediate solution to determine the number of states leading to the results and the inclusion conditions of these states, and then use the results of the parsimonious solution to determine the core conditions that are more important for a given set of states (Du and Jia, 2017; Zhang and Liu, 2023). Therefore, only the parsimonious and intermediate solutions will be reported in this study, and thus, the complex solutions will not be included.

Table 4 depicts the results from the intermediate and parsimonious solutions obtained through fsQCA. The results reveal six solutions that affect the compensation incentive effect, with an overall solution consistency of 0.916, which is greater than the threshold of 0.8. This indicates that these configurations are sufficient conditions for compensation incentives and there is agreement. In addition, the overall solution coverage was 0.429, indicating that these solutions explained 42.9% of the cases of compensation incentives. Analysis reveals that these six solutions affecting the effect of compensating incentives can be grouped into two configurations.

**Table 4:** Results of the analysis of truth table.

							
		H1a	H1b	H1c	H2a	H2b	H2c
ζ1	X1	●	●	●	●	●	●
	X2	●	●	⊗	●	●	●
	X3	●	●		●	●	●
ζ3	X6	⊗		●	●	●	●
	X7	⊗	⊗	●	●	●	●
	X8		●	●	●		●
ζ4	X10	●	●	●	●	●	
	X11	●	●	●			●
	X12	●	●	●	●	●	●
Consistency		0.919	0.951	0.946	0.957	0.957	0.949
Raw coverage		0.247	0.256	0.253	0.298	0.302	0.326
Unique coverage		0.025	0.004	0.035	0.008	0.004	0.036
Solution consistency				0.916			
Solution coverage				0.429			

●, presence of core conditions; ●, presence of peripheral conditions; ⊗, absence of peripheral conditions.

Configuration 1 involves the “economic-value” pattern. H1a, H1b, and H1c have the core conditions X1, X10, X11, and X12, so the three configurations are grouped. It emphasizes paying attention to the direct economic compensation such as athletes’ basic income, allowances, and subsidies, and indirect non-economic compensation such as career achievement, training autonomy, and development opportunities, which reflect the value of the work.

Among the differences is that H1a is joined with X3 as the core condition and X2 as the marginal condition (consistency of 0.919, raw coverage of 0.247). It also shows that, regardless of whether there are suitable training conditions or not, when the social status of athletes and the attention of leaders are insufficient, as long as athletes’ direct economic compensation such as basic salary, allowance and subsidy, and indirect non-economic compensation reflecting the value of the work such as career achievements, training autonomy, development opportunities, etc., are fulfilled, it can also increase the motivation of athletes to train and enhance the effect of incentives. It is very close to H1b and H1a, with the nuance that the Training condition, which has no effect in H1a, is marginal in H1b (Consistency = 0.951, Raw Coverage = 0.256). It suggests that, regardless of social status, enhancing athletes’ basic salary and allowance supplements, strengthening ideological education for athletes to enhance Career achievement and Training autonomy, and strengthening athletes’ vocational training to increase diversified career development opportunities can also enhance the effectiveness of motivation when leadership attention is insufficient. The H1c adopts X1, X6, X10, X11, and X12 as the core condition, and X2, ~X7, X8 as the marginal conditions (Consistency = 0.946, Raw Coverage = 0.253). It shows that, regardless of whether there are allowance supplements or not, as long as the basic income of the athletes is guaranteed, the training environment of the athletes is optimized (e.g., social status, leadership attention, training condition), and the elements of the athletes’ professional values are paid attention to, such as Career achievement, training autonomy, development opportunity, etc., so that even if there is a lack of bonus income, the training enthusiasm of athletes can be improved and the incentive effect can be enhanced.

Configuration 2 refers to the “Economic-environmental-value” equilibrium. Additionally, H2a, H2b, and H2c are based on the core conditions of X1, X3, X6, and X12, which emphasize the optimization of athletes’ economic income, social status, and career development opportunities and other related factors to enhance the effect of athletes’ compensation incentives.

The difference is that H2a is adding X3 as the core condition and presenting X2, X7, and X8 as the marginal conditions (Consistency = 0.957, Raw Coverage = 0.298). Results show that H2b is very close to H2a, with the difference being that the marginal training condition in H2a does not affect H2b (Consistency = 0.957, Raw Coverage = 0.302). It also shows that regardless of whether athletes have training autonomy or not, focusing on economic elements such as basic income, allowances, and subsidies, and non-economic elements such as improving social status, career fulfillment, and career development opportunities, as well as optimizing the proportion of athletes’ bonus income and strengthening the importance of leadership, can also improve the effect of athletes’ compensation incentives. Furthermore, H2c takes X1, X3, X6, X11, and X12 as the core conditions and X2, X7, and X8 as the marginal conditions (Consistency = 0.949, Raw Coverage = 0.326). It suggests that regardless of athletes’ career fulfillment, athletes can also be motivated to train as long as they can be assured of financial income, an external training environment, and opportunities for career development.

**Figure 3:**
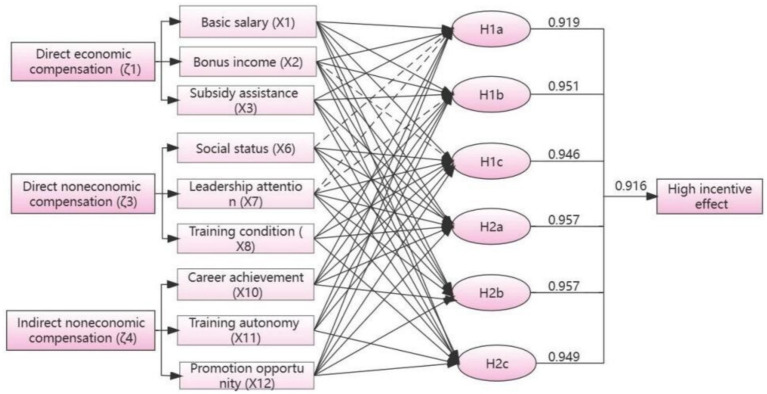
Results of six configurations.

The results of the six configurations were plotted, and it can be seen from Figure 3 that the antecedent conditions of direct economic compensation, direct non-economic compensation, and indirect non-economic compensation existed in five of the configurations. Thus, to improve the motivational effect on athletes, it is necessary to improve the three aspects together, which also confirms the validation results of the structural equation modeling. Among the six configuration results, the antecedent conditions of basic income and career development opportunities appeared six times overall, which indicates the importance of these two elements for the influence of athletes’ motivational effects. Therefore, it is necessary to carry out sufficient market investigation first, link the basic salary of athletes with the local minimum wage line, to protect the basic needs of athletes, based on optimizing the training of athletes’ career development, promoting the comprehensive development of athletes’ qualities, and strengthening the retirement protection of athletes, to enhance the incentive effect of athletes’ compensation.

### 4.3 Robustness test results

Generally speaking, there are three main ways to validate the robustness of QCA methods: by replacing the calibration anchors, by adjusting the frequency of sample cases, and by raising the consistency threshold (Ou et al., 2021). In this paper, the consistency threshold is adjusted upward to 0.85, and the increase of the consistency value of PRI has no substantial effect on the number of groupings and the distributional arrangement of the core and edge conditions, and the new groupings are consistent with the original groupings, which illustrates that the findings of this paper are robust and reliable.

## 5 Conclusion and contributions

### 5.1 Research conclusion

The study attempts to use a mixed method of quantitative analysis (SEM) and qualitative analysis (fsQCA) to analyze the net and combination effects of athletes’ direct economic compensation, indirect economic compensation, direct non-economic compensation, and indirect non-economic compensation on the incentive effect, revealing the mechanism of each element of compensation on the incentive effect of athletes’ compensation. The conclusions drawn from the structural equation modeling can show that athletes’ direct economic compensation, indirect non-economic compensation, and direct non-economic compensation have a significant positive effect on the incentive effect of compensation, and indirect economic compensation satisfaction does not have a significant effect on the incentive effect of athletes’ compensation. On this basis, three latent variables (ζ_1_, ζ_3,_ and ζ_4_) and nine observed variables (X1, X2, X3, X6, X7, X8, X10, X11, and X12) affecting the effect of compensation incentives are grouped and analyzed, and the combined effect of the linkage of the conditional variables is considered. Furthermore, the FsQCA analysis results show that five of the six groups of states to enhance the incentive effect of athletes’ compensation have antecedent conditions such as direct economic compensation, direct non-economic compensation, and indirect non-economic compensation, so to enhance the incentive effect of athletes, it is necessary to improve the incentive effect of athletes from the three aspects together. This also confirmed the validation results of the structural equation modeling.

### 5.2 Research contribution

The study creates a comprehensive theoretical model based on the compensation structure and reveals the internal process of the various elements of compensation affecting the incentive effect of athletes. Also, by combining quantitative and qualitative analyses, it not only reveals the intrinsic causality between the variables but also provides a more in-depth analysis of the antecedent conditions of the incentive effect of athletes’ compensation and discovers many equivalent paths to enhance the incentive effect. It also provides practical insights for managers in the sports sector to fully understand the impact of the various elements of compensation, and to establish multiple combinations of incentive models with “basic salary & career achievement & training autonomy & promotion opportunities” and “basic salary & subsidy assistance & social status & promotion opportunity” as the core conditions, within the conditions of limited resources. Although athletes consider the spiritual level very important and aspire to be respected by the organization and recognized by society, basic material needs are also essential. Therefore, there is a need to optimize career development planning for athletes and their professional reputation while continuously improving their basic salary structure.

### 5.3 Research limitations

There are also some limitations to this study. Future research should explore other conditions (e.g., compensation equity perceptions, work-life balance, etc.) that influence the compensation incentive effects of athletes. Finally, the number and types of athletes should be enriched to explore the individual variability of factors influencing compensation incentive effects.

## Data Availability

The raw data supporting the conclusions of this article will be made available by the authors, without undue reservation.
